# The role of socio-demographic factors in premature cervical cancer mortality in Colombia

**DOI:** 10.1186/s12889-016-3645-1

**Published:** 2016-09-15

**Authors:** Silvia Bermedo-Carrasco, Cheryl L. Waldner

**Affiliations:** 1 School of Public Health, University of Saskatchewan, 104 Clinic Place, Saskatoon, SK S7N 5E5 Canada; 2 Western College of Veterinary Medicine, University of Saskatchewan, 52 Campus Drive, Saskatoon, SK S7N 5B4 Canada

**Keywords:** Uterine cervical neoplasms, Mortality, Colombia, Socioeconomic factors, Inequities

## Abstract

**Background:**

While cervical cancer (CC) is an important cause of premature mortality in Colombia, the impact of socio-demographic factors on CC mortality in young women is not well understood. The primary objective of this study was to identify differences in CC mortality among Colombian women aged 20–49 years associated with education, type of health insurance, urban or rural and region of residence, and to determine whether differences in mortality associated with education or insurance varied by age.

**Methods:**

Cervical cancer deaths for 2005–2013 and risk factors were obtained from the National Administrative Department of Statistics. Populations at risk were calculated from age-stratified population projections and the 2010 National and Demographic Health Survey. Negative binomial regression models, stratified by age, were used to examine associations between socio-demographic factors and mortality rates and whether the effects of education and health insurance varied by age. Multiple imputation was used to examine the importance of missing data.

**Results:**

Differences of CC mortality were identified among women with limited to no education compared to highly educated women, with the largest disparity in the youngest age group (IRR 26.8, 95 % CI 6.65–108). Differences in mortality associated with health insurance also varied based on age group. Women with contributory and special health insurance had lower mortality rates than women with subsidised or no health insurance, except in the youngest age group. No differences were observed between women with subsidised and those with no insurance in any age group. Mortality rates were high among women who resided in urban areas and in the Atlantic, Central, Pacific, and Amazon-Orinoquía regions of Colombia. Missing values in the mortality database did not impact the findings from this study.

**Conclusions:**

Limited education was most strongly associated with premature CC mortality in the youngest women. Subsidised insurance did not appear to provide significant protection against CC mortality when compared to not having insurance, suggesting the need to examine diagnostic and treatment services available under the subsidised insurance plan. Our results could be used to target interventions to optimise the impact of resources to prevent premature mortality due to CC in Colombia.

**Electronic supplementary material:**

The online version of this article (doi:10.1186/s12889-016-3645-1) contains supplementary material, which is available to authorized users.

## Background

Cervical cancer (CC) imposes a high burden of disease worldwide, being the third most important cause of cancer-related deaths among women in 2012 [1]. Developing countries account for almost 90 % of total CC deaths [1]. Developing countries are further inequitably impacted by premature CC deaths in young women [2], including mothers and caregivers [3] and, in many cases, important contributors to family income [4]. In Latin American countries, CC caused more than 28,000 female deaths in 2012 [1]. Compared to Canada and the United States, Latin American countries have greater age-standardised mortality rates due to CC, especially in Central America and countries located in the Andean region [5]. In Colombia, a country part of the Andean region, CC has been ranked as the second most common cause of cancer deaths in women after breast cancer [6].

While mortality rates from CC in Colombia have been decreasing in recent years [7], the burden of this disease continues to be an important concern [6], in spite of having effective tools for prevention [2]. Measured as total avoidable years of life lost in Colombia, CC ranks above other causes of mortality, such as hypertensive heart disease or liver cancer [8]. Because cancer in Colombia is often diagnosed in late stages, the effectiveness of potential treatment options can be limited [9]. Additionally, many people in Colombia do not have health insurance, even though they can be affiliated with the contributory or subsidised system depending on their capacity to pay [10]. Public teachers, university workers, police or military forces, and employees of the Colombian Oil Company have special health insurance plans [10].

Previous studies in Colombia have described associations between one or two socio-demographic factors and CC mortality focused on wider age ranges, including all women more than 15 years of age [11, 12] and women aged 25 to 64 years [13]. Differences in CC mortality have been explored among departments, or Colombian administrative divisions [11, 14], rural or urban residence [11], educational level [12, 13], and lack of health insurance [14]. However, there is no evidence regarding how these socio-demographic variables impact premature mortality associated with CC in young women (i.e., women aged 20–49 years) in Colombia, or how these risk factors might differentially impact younger as compared to older women under 50 years of age. The need for studies centred on young women to better understand specific risk factors for premature deaths from CC has previously been identified [2].

Nationwide studies are needed to understand the specific roles of education and type of health insurance in CC mortality among young women in Colombia, while accounting for differences between urban and rural residences and variation across geographic regions. Moreover, variations in CC mortality between limited-to-no-educated and highly educated women by age group need to be explored, given that Colombian women 25 years or more tend to make more use of their rights to access health care [15] and that young women have low quality of reproductive and sexual health [16]. The ability of women to act on the information they gain from their education might vary based on age.

Similarly, age-specific differences in the effect of type of health insurance as a risk factor for CC mortality should be considered because most Colombians who use the *tutela* action (i.e., a legal constitutional mechanism to protect fundamental human rights [17]) to access health services had contributory health insurance [15] and older age is related to an increased utilisation of health care [18]. As for education, the capacity of women to access the services available under their health insurance might vary based on their age.

The resulting information from nationwide studies considering these variations could be used to identify targets for intervention in the diagnosis and treatment of young women with CC, taking into consideration the effect of CC on young women [2], as well as the existence of marked regional [19], health care system-related [20], and educational disparities in the Colombian population [19].

The study described here examined differences associated with socio-demographic factors in CC mortality among young women in Colombia between 2005 and 2013. The objectives of this study were to: 1) describe socio-demographic characteristics of women aged 20–49 years who died from CC, 2) identify differences in CC mortality rates by educational level, type of health insurance, urban or rural residence, and geographic region of residence among women aged 20–49 years, and 3) evaluate if there were age-specific differences in the importance of education or type of insurance as risk factors for CC mortality.

## Methods

### Source of cervical cancer mortality data stratified by potential risk factors

Official mortality records of all individuals who died in Colombia between January 2005 and December 2013 were obtained from the National Administrative Department of Statistics (*Departamento Administrativo Nacional de Estadística*—DANE). The causes of death in these records were coded according to the International Classification of Diseases—10^th^ revision. The code C53 (malignant neoplasm of cervix uteri), along with applicable sub-codes (C530, malignant neoplasm of the endocervix; C531, malignant neoplasm of the exocervix; C538, overlapping lesion of cervix uteri; C539, cervix uteri, unspecified) were used to extract all female deaths attributed to CC by year of death. Additionally, unspecified malignant neoplasms of the uterus (code C55) were reallocated according to the proportion of deaths due to cervical (code C53) and corpus uterine cancer (code C54) by age group and year of death, as recommended by Loos et al. [21].

All female deaths from CC were consolidated in one data set. The socio-demographic characteristics of each woman, including age, educational level, type of health insurance, rural or urban residence, and geographic region of residence, were then extracted from the mortality records to be considered as potential risk factors in the analysis. From this data set, the subset of women aged 20–49 years was selected for analysis. The total numbers of observed CC deaths were stratified by age: 20–24 years, 25–29 years, 30–34 years, 35–39 years, 40–44 years, and 45–49 years. The resulting outcome for the analysis was the age-group specific count of deaths due to CC further stratified by one or more of the following variables: educational level, type of health insurance, urban or rural residence, and department of residence. Department CC counts per age group were summarised for each of the five geographic regions described in the 2010 National and Demographic Health Survey (NDHS) [22]. The NDHS evaluated different factors associated with reproductive and sexual health in a sample of more than 53,000 women between 13 and 49 years.

Mortality data used for this analysis were publicly available upon request to DANE and, therefore, this study was exempted from ethics review by the University of Saskatchewan Ethics Board.

### Source of population at risk data stratified by potential risk factors

The numbers of women at risk of dying due to CC for the risk factor-specific strata were extracted from population projections by DANE [23] and the NDHS data sets.

In the first step, 2009 national population projections based on the 2005 census [23] were used to determine the population at risk categorised by the same five-year age groups used for CC cases. The population at risk was based on 2009 information as this was the mid-point of the 2005 to 2013 study period. Total department counts of women at risk per age group were classified in one of the five geographic regions used in the NDHS.

In the second step, women at risk were stratified based on the other socio-demographic variables of interest. The proportions of women between 20 and 49 years of age for each level of education, type of health insurance, and for urban or rural residence were calculated from the NDHS data set for each 5-year age group and region of residence. The appropriate proportions were then applied to the 2009 population projections for each age group and region of residence to generate the necessary strata-specific numbers of at-risk women for subsequent analyses.

### Statistical analysis

The total numbers and proportions of CC deaths were described for each category of the risk factors of interest using all available data. The same descriptors were calculated for the subset of cases with complete information for all potential risk factors of interest. The subsequent analysis was completed using two different analytical approaches to evaluate the importance of missing risk factor information in the DANE mortality records. The first approach excluded women who had unavailable or missing information in any of the variables of interest (complete case analysis). The second approach recognised the potential for selection bias by excluding cases with missing data and applied multiple imputation methods with the choice of technique informed by the missing data patterns [24]. All the analyses were completed in STATA version 13 (StataCorp LP, College Station, TX, USA).

#### Complete case analysis

The first analysis considered only women aged 20–49 years with complete information for all of the variables of interest. Associations between each potential risk factor and CC mortality stratified by age group were individually evaluated using negative binomial models. The natural log of the population at risk stratified by age group and each risk factor was used as the offset in these regression models. Risk factors with *p*-values <0.2 were considered for inclusion in the multivariable analysis [25]. A Wald test was used to estimate the overall *p*-value for multi-category variables. A likelihood ratio test was used to compare the negative binomial to the Poisson model [26]. Preliminary analysis suggested that a negative binomial distribution fit the data better than that a Poisson distribution.

Multivariable negative binomial regression models were then used to identify differences in CC mortality, first by educational level (Model 1) and then by type of health insurance (Model 2). The decision was made to create two separate models because of the potential for type of health insurance to be an intervening variable on the causal pathway between educational level and CC mortality. Better education could lead to better insurance which then results in lower CC mortality. Correcting for insurance could result in biased underestimates of the direct impact of education on CC mortality [25].

Both models were analysed using the same set of variables (i.e., age group, region of residence, and urban or rural residence) to control for potential confounders. Interactions between age and educational level, as well as age and type of health insurance were evaluated. Pairwise comparisons were used to examine differences in CC mortality among categories of education for each age group and across categories of insurance for each age group.

A third model simultaneously evaluating all independent variables of interest intended to measure the joint effect of the education and health insurance did not converge‚ given that stratification of the population at risk resulted in denominators with zero counts. When the cells with zero denominators were eliminated, the model did converge, but 15 % of the outcome observations were lost introducing a risk for selection bias. Interactions were not examined in this model. The results of this model were compared to main effects only models with education and then with insurance.

#### Imputed data analysis

The second approach to the analysis applied multiple imputations to minimise potential biases and loss of power and precision associated with missing risk factor data in the DANE mortality files. The patterns of missingness were visually assessed using the *misstable* command in STATA to determine an appropriate method for imputation [24]. The result was a table showing the percentage of data with various patterns of missingness according to each of the variables. Variables were marked as missing or not missing for a given pattern. Multiple imputation by chained equations was chosen to optimise the analysis of the socio-demographic factors of interest [27], based on the percentage of all women, including those with incomplete data [28], who died from CC.

The method recommended by van Buuren et al. [29] was followed to specify the multiple imputation model. This model incorporated data from all females who died from CC between 2005 and 2013 to account for those with missing age and included the socio-demographic variables of interest, as well as auxiliary variables. The auxiliary variables considered included: urban or rural area where the death occurred, facility or place of death (e.g., home, health centre, hospital, etc.), marital status, person who certified the death, and year and region of death.

Using the mean frequency of the imputed data, women aged 20–49 years stratified by age groups were again cross-classified according to the risk factors of interest to obtain the number of strata-specific CC deaths. Negative binomial models were used to evaluate relationships between each socio-demographic factor and CC mortality stratified by age, with the corresponding population at risk used to determine the offset as previously described. Risk factors with *p*-values <0.2 were considered for inclusion in the multivariable analysis [25].

Two multivariable negative binomial regression models were used to identify differences in CC mortality by educational level (Model 1) and type of health insurance (Model 2), as described for the complete case analysis. The multiple imputation models also accounted for age group, region of residence, and urban or rural residence. As described for the complete case analysis, the interactions between age and education and age and type of insurance were evaluated.

Results were reported as incidence rate ratios (IRR) and 95 % confidence intervals (95 % CI). Differences were considered statistically significant if *p* < 0.05. Comparisons between models were done used the Akaike’s information criterion (AIC) [25].

## Results

From 2005 to 2013, 1,768,273 deaths were reported in Colombia; 756,636 were women of any age. During this period, 14,355 women died from CC (code C53 and applicable sub-codes) and 2535 were classified as unspecified malignant neoplasms of the uterus (code C55). From the unspecified category, 2296 (90.6 %) cases were reallocated to CC and 239 (9.4 %) to corpus uterine. Therefore, the number of females of all ages who died from CC was 16,651, which corresponded to 2.2 % of all deaths in females of all ages during the study period. Seventeen women in this group were eliminated from the data set because they resided out of the country, resulting in 16,634 women who died due to CC and resided in Colombia in 2005–2013.

From the 16,634 CC cases (excluding 18 cases with missing age), 5093 women were aged 20–49 years, representing 30.6 % of all deaths due to CC. The mean age of this group was 40.5 years (SD = 6.4). Most women had primary education and subsidised health insurance (Table 1). A third of women lived in the Eastern region and most resided in urban areas of Colombia.

**Table 1:** Socio-demographic characteristics of women who died from cervical cancer and cases with complete data

	Women 20–49 years			
Socio-demographic characteristics	Total cervical cancer mortality (*n* = 5093)		Cervical cancer mortality with complete data (*n* = 4247)	
	n	(%)^a^	n	(%)^a^
Educational level				
No education	346	(6.8)	334	(7.9)
Primary	2194	(43.1)	2136	(50.3)
Secondary	1506	(29.6)	1486	(35.0)
Higher	294	(5.8)	291	(6.9)
Missing information	753	(14.8)	–	–
Type of health insurance				
Non-affiliated	620	(12.2)	497	(11.7)
Subsidised	2863	(56.2)	2359	(55.5)
Special	86	(1.7)	77	(1.8)
Contributory	1435	(28.2)	1314	(30.9)
Missing information	89	(1.7)	–	–
Urban or rural residence				
Rural	829	(16.3)	687	(16.2)
Urban	4204	(82.5)	3560	(83.8)
Missing information	60	(1.2)	–	–
Region of residence				
Atlantic	1053	(20.7)	831	(19.6)
Central	1353	(26.6)	1164	(27.4)
Pacific	959	(18.8)	826	(19.4)
Amazon-Orinoquía	167	(3.3)	130	(3.1)
Eastern	1553	(30.5)	1296	(30.5)
Missing information	8	(0.2)	–	–
Age groups				
20–24 years	64	(1.3)	56	(1.3)
25–29 years	273	(5.4)	227	(5.3)
30–34 years	630	(12.4)	541	(12.7)
35–39 years	1040	(20.4)	872	(20.5)
40–44 years	1391	(27.3)	1154	(27.2)
45–49 years	1695	(33.3)	1397	(32.9)

Women aged 20–49 years who died due to cervical cancer in Colombia between 2005 and 2013. The table summarises all available data for women who died from cervical cancer and data for women with complete data for age, region of residence, educational level, type of health insurance, and rural or urban residence

^a^Percentage of total cases in each category

### Complete case analysis

Of the 5093 women who died from CC, 4247 (83.4 %) had complete data for all risk factors of interest (Table 1). The negative binomial models, stratified by 5-year age category, identified significant differences in CC mortality among educational levels (Wald test, *p* < 0.0001), types of health insurance (Wald test, *p* < 0.0001), urban or rural residence (*p* < 0.0001), and region of residence (Wald test, *p* < 0.0001). Age by itself was also significantly associated with CC mortality (Wald test, *p* < 0.0001).

**Fig. 1:**
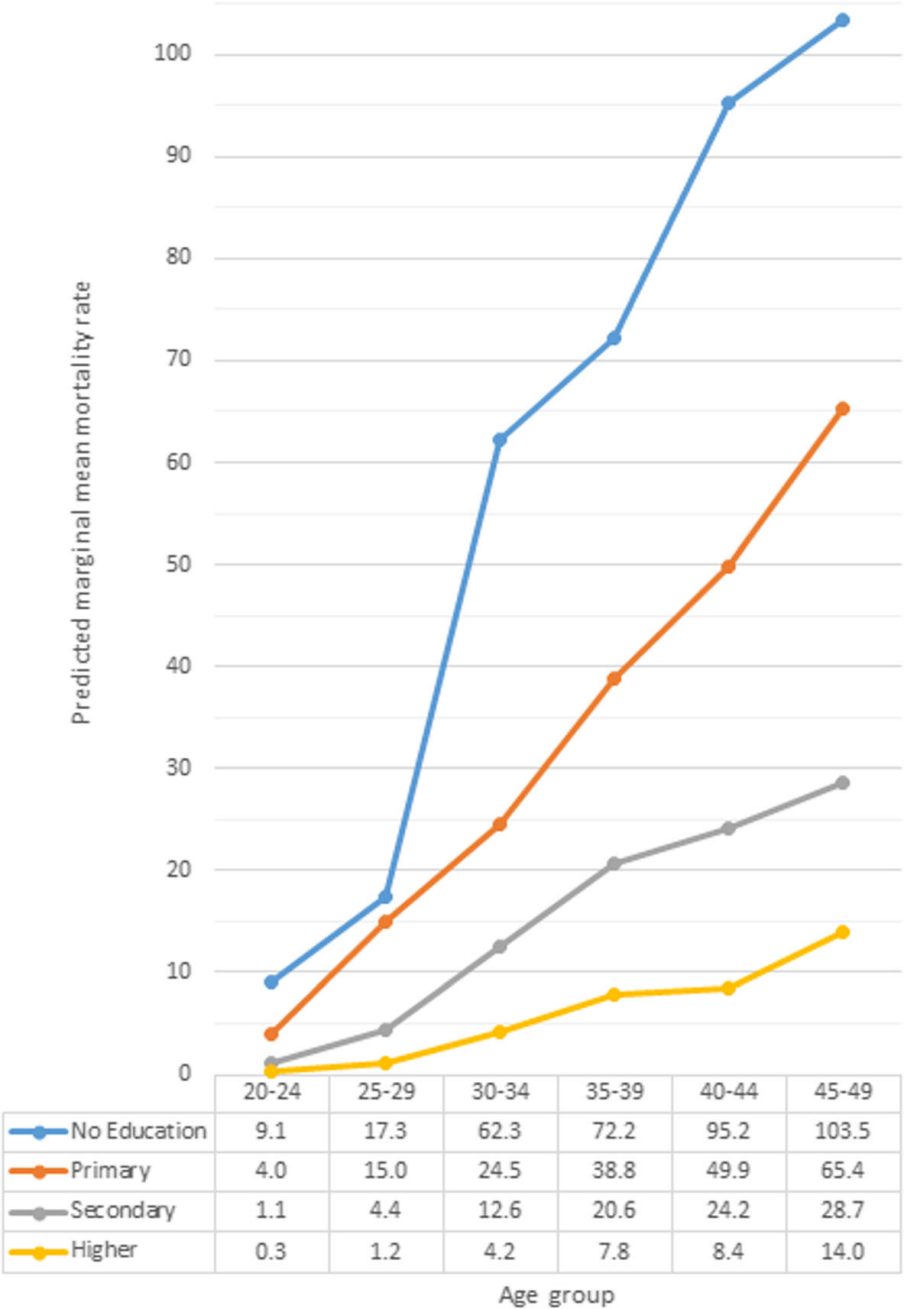
Marginal mean mortality rates due to cervical cancer according to age groups and educational level of women. Mortality rates presented here are adjusted by rural or urban residence and region of residence

The final model describing the association between education and CC mortality included rural or urban residence, region of residence, and age is presented in Tables 2, 3 and 4 and Additional file 1. A significant interaction was detected between educational levels and age groups (Wald test, *p* < 0.0001) (Fig. 1, Table 2 [Model 1], and Table 3). Differences in CC mortality were observed among women with limited or no education compared to women with higher education across all age groups. However, the relative size of these differences tended to be larger among younger women than for those in the oldest age group (Table 2 [Model 1]). For example, when comparing women with primary education to those with higher education, the IRR for women aged 25–29 was significantly higher than the IRR for women aged 45–49 years based on non-overlapping confidence intervals. Larger differences in education also tended to be associated with higher IRR for all age groups than smaller differences in education.

**Table 2:** Effect estimates for interacting variables in the cervical cancer models limited to complete data (*n* = 4247)

	Associations between educational level or type of health insurance and cervical cancer mortality for each age group (years)											
	20–24		25–29		30–34		35–39		40–44		45–49	
	IRR	(95 % CI)	IRR	(95 % CI)	IRR	(95 % CI)	IRR	(95 % CI)	IRR	(95 % CI)	IRR	(95 % CI)
Model 1												
Educational level												
No education vs. higher education	32.5	(7.70–137)	14.3	(6.14–33.5)	14.7	(9.30–23.2)	9.27	(6.30–13.6)	11.4	(7.96–16.3)	7.42	(5.42–10.1)
Primary vs. higher education	14.1	(5.27–38.0)	12.4	(7.34–21.0)	5.77	(4.08–8.14)	4.98	(3.69–6.73)	5.96	(4.37–8.13)	4.69	(3.58–6.13)
Secondary vs. higher education	3.87	(1.47–10.2)	3.62	(2.13–6.14)	2.97	(2.10–4.21)	2.65	(1.94–3.60)	2.89	(2.10–3.97)	2.06	(1.54–2.75)
No education vs. primary education	2.30	(0.68–7.77)	1.15	(0.56–2.40)	2.55	(1.75–3.71)	1.86	(1.37–2.53)	1.91	(1.49–2.45)	1.58	(1.26–1.99)
Model 2												
Type of health insurance												
No insurance vs. contributory insurance	1.50	(0.60–3.72)	2.18	(1.43–3.35)	1.54	(1.12–2.13)	1.48	(1.12–1.94)	1.57	(1.21–2.03)	2.21	(1.73–2.81)
Subsidised vs. contributory insurance	1.96	(0.99–3.85)	1.79	(1.27–2.51)	1.74	(1.36–2.21)	1.55	(1.25–1.91)	1.96	(1.61–2.39)	1.96	(1.63–2.37)
Special vs. contributory insurance	2.91	(0.64–13.1)	0.29	(0.04–2.10)	0.64	(0.30–1.39)	0.59	(0.32–1.10)	0.93	(0.61–1.42)	0.79	(0.52–1.19)
Subsidised vs. special insurance	0.67	(0.16–2.82)	6.17	(0.86–44.4)	2.71	(1.26–5.81)	2.61	(1.41–4.83)	2.10	(1.40–3.16)	2.49	(1.6–3.74)
No insurance vs. special insurance	0.51	(0.11–2.44)	7.54	(1.03–55.2)	2.40	(1.09–5.31)	2.49	(1.31–4.73)	1.68	(1.08–2.61)	2.80	(1.82–4.32)
No insurance vs. subsidised insurance	0.77	(0.35–1.68)	1.22	(0.83–1.80)	0.89	(0.66–1.20)	0.95	(0.74–1.23)	0.80	(0.63–1.02)	1.12	(0.90–1.40)

*IRR* Incidence rate ratios, *95 % CI* 95 % confidence intervals

Model 1 assessed differences in cervical cancer mortality rates by educational level and Model 2 evaluated differences in mortality rates by type of health insurance. Both multivariable models included fixed effects for age group, urban or rural residence, and region of residence, as well as interactions with age. Only women with complete data for the risk factors of interest were included in these analyses

**Table 3:** Effect estimates for the interaction between age and education from complete and imputed data models

	Associations between age group and cervical cancer mortality for each level of education							
	No education		Primary education		Secondary education		Higher education	
Age groups (years)	IRR	(95 % CI)	IRR	(95 % CI)	IRR	(95 % CI)	IRR	(95 % CI)
Complete data analysis (*n* = 4247)								
25–29 vs. 20–24	1.90	(0.50–7.21)	3.78	(2.32–6.16)	4.03	(2.56–6.34)	4.31	(1.58–11.8)
30–34 vs. 20–24	6.83	(2.09–22.3)	6.16	(3.86–9.82)	11.6	(7.57–17.8)	15.1	(5.93–38.5)
35–39 vs. 20–24	7.90	(2.46–25.4)	9.77	(6.18–15.4)	19.0	(12.4–28.9)	27.7	(11.0–69.9)
40–44 vs. 20–24	10.4	(3.28–33.1)	12.6	(7.97–19.8)	22.2	(14.6–33.8)	29.8	(11.8–75.5)
45–49 vs. 20–24	11.3	(3.58–35.9)	16.5	(10.5–25.9)	26.4	(17.3–40.4)	49.7	(19.9–124)
Imputed data analysis (*n* = 5098)								
25–29 vs. 20–24	2.61	(0.72–9.40)	3.85	(2.47–6.02)	4.46	(2.90–6.87)	4.19	(1.67–10.5)
30–34 vs. 20–24	7.55	(2.32–24.6)	6.28	(4.09–9.63)	12.5	(8.33–18.9)	13.8	(5.85–32.6)
35–39 vs. 20–24	10.0	(3.14–32.0)	10.1	(6.65–15.4)	20.6	(13.7–30.8)	25.4	(10.9–59.2)
40–44 vs. 20–24	13.3	(4.21–42.1)	12.9	(8.52–19.6)	24.1	(16.1–36.0)	28.9	(12.4–67.6)
45–49 vs. 20–24	15.0	(4.76–47.3)	16.8	(11.1–25.4)	29.4	(19.6–44.0)	47.4	(20.5–110)

*IRR* Incidence rate ratios, *95 % CI* 95 % confidence intervals

Results summarise both the analysis of data for cases with complete information on all risk factors of interest and the imputed data analysis for models examining the association between educational level and cervical cancer mortality (Model 1). Both multivariable models included fixed effects for age group, urban or rural residence, and region of residence, as well as interactions between educational level and age

**Table 4:** Effect estimates for non-interacting variables from of cervical cancer mortality models with complete data (*n* = 4247)

	Associations with cervical cancer mortality			
	Model 1 Educational level		Model 2 Type of health insurance	
	IRR	(95 % CI)	IRR	(95 % CI)
Urban or Rural residence				
Rural	0.39	(0.35–0.43)	0.52	(0.47–0.57)
Urban	Ref.		Ref.	
Region of residence				
Atlantic	1.13	(1.00–1.28)	1.04	(0.92–1.18)
Central	1.30	(1.15–1.47)	1.28	(1.14–1.44)
Pacific	1.39	(1.23–1.57)	1.34	(1.18–1.51)
Amazon-Orinoquía	1.61	(1.32–1.97)	1.64	(1.34–2.01)
Eastern	Ref.		Ref.	

*IRR* Incidence rate ratios, *95 % CI* 95 % confidence intervals

Model 1 assessed differences in cervical cancer mortality rates by educational level and Model 2 evaluated differences in mortality rates by type of health insurance. Both multivariable models included fixed effects for age group, urban or rural residence, and region of residence, as well as interactions with age. Only women with complete data for the risk factors of interest were included in these analyses

Furthermore, across all education levels, women in older age groups tended to have higher mortality rates than those in the youngest age group (Fig. 1 and Table 3). The relative size of differences in risk associated with increasing age also tended to be smaller for women with less education compared to women with more education.

Table 4 shows the effect estimates for variables that were not included in the interaction between age and educational level in the multivariable model (Model 1). After adjusting for age group, region of residence, and urban or rural residence, CC mortality rates were lower among women from rural compared to those from urban areas. Mortality rates for women who resided in the Atlantic, Central, Pacific, and Amazon-Orinoquía regions were higher than those for women from the Eastern region.

The second multivariable model evaluating the effect of type of insurance showed similar results for rural and urban differences and region of residence to Model 1 (Table 4 [Model 2] and Additional file 2). However, in this model, there were no differences in CC mortality rates between women from the Atlantic and the Eastern regions.

**Fig. 2:**
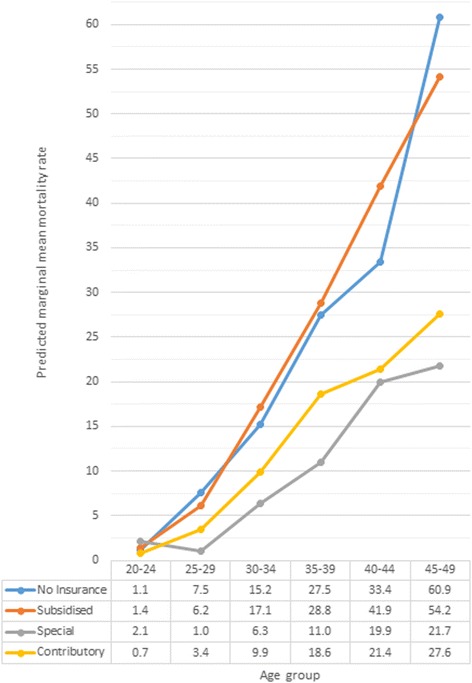
Marginal mean mortality rates due to cervical cancer according to age groups and type of health insurance of women. Mortality rates presented here are adjusted by rural or urban residence and region of residence

The second multivariable model also included a significant interaction between the type of health insurance and age group (Wald test, *p* < 0.0001) (Table 2 [Model 2] and Additional file 2). Mortality rates from CC were higher among women with no insurance and subsidised insurance compared to women with contributory insurance, except for women aged 20–24 years (Fig. 2 and Table 2 [Model 2]). Also, differences in CC mortality rates were observed between women with subsidised and special insurance among women aged 30+ years. Furthermore, mortality rates for women with no insurance were higher than women with special insurance, except for those aged 20–24 years. There were no significant differences between women with no insurance and subsidised insurance in any age group, nor between special and contributory insurance in any age group.

The model including type of health insurance (Model 2 and Additional file 2) explained a larger portion of the total variance in CC mortality (AIC = 1099) than the model including level of education (Model 1) (AIC = 1136) (Additional file 1).

In a subset model (*n* = 4234) examining the simultaneous associations of educational level and type of health insurance adjusted for region of residence and rural or urban residence, both education (Wald test, *p* < 0.0001) and type of health insurance (Wald test, *p* = 0.02) were associated with mortality due to CC. For example, the mortality rate for women with no education was higher than that for women with higher education (IRR = 9.25, 95 % CI 7.56–11.31) after adjusting for type of insurance and other risk factors. This was similar to the effect resulting from a separate model with educational level (IRR = 10.5, 95 % CI 8.68–12.70) adjusted for all other risk factors except type of insurance but with no interaction term. Also, the mortality rate for women with no health insurance was higher than for women with contributory insurance (IRR = 1.16, 95 % CI 1.02–1.33) in the model adjusted for education and other risk factors. This result was similar to that obtained using a separate model for type of health insurance (IRR = 1.74, 95 % CI 1.53–1.99) adjusted for all other risk factors except education but with no interaction term.

### Missing data, multiple imputation process, and imputed data analysis

Most women of all ages who died from CC (*n* = 16,634) had complete information in the socio-demographic factors of interest and auxiliary variables (80.3 %). Among the variables of interest, there were missing values for age (0.1 %), educational level (16.3 %), type of health insurance (1.8 %), rural or urban residence (1.0 %), and region of residence (0.2 %). On the other hand, among the auxiliary variables, there were missing values for urban or rural area of death (0.2 %), facility or place of death (0.2 %), and marital status (7.0 %). The visual assessment of the missing data in the variables of interest, showed a random missing or non-systematic pattern, in which the missing values had no special order or distribution. Taking into consideration that 19.7 % of the women of all ages had one or more missing values, 20 imputations by chained equations were computed.

Using the imputed data for all women who died from CC, 5098 cases were between 20 and 49 years. This number included five women whose age was missing in the original data and was then imputed within the age range of study. The two final multivariable models for CC mortality using the imputed data included fixed effects for age, rural or urban area of residence, region of residence, and an interaction with age in addition to either level of education or type of insurance (Tables 5 and 6, Additional files 3 and 4). The effect estimates for the variables of interest that were not interacting for the two models shown in Table 5 had the same direction and similar effect sizes of the estimates resulting from the complete case analysis (Table 4).

**Table 5:** Effect estimates for non-interacting variables resulting from the models with imputed data (*n* = 5098)

	Associations with cervical cancer mortality			
	Model 1 Educational level		Model 2 Type of health insurance	
	IRR	(95 % CI)	IRR	(95 % CI)
Urban or Rural residence				
Rural	0.39	(0.36–0.43)	0.52	(0.47–0.57)
Urban	Ref.		Ref.	
Region of residence				
Atlantic	1.20	(1.08–1.34)	1.10	(0.98–1.23)
Central	1.25	(1.12–1.40)	1.24	(1.11–1.39)
Pacific	1.34	(1.19–1.49)	1.28	(1.14–1.43)
Amazon-Orinoquía	1.68	(1.41–2.01)	1.74	(1.45–2.09)
Eastern	Ref.		Ref.	

*IRR* Incidence rate ratios, *95 % CI* 95 % confidence intervals

Model 1 assessed differences in cervical cancer mortality rates by educational level and Model 2 evaluated differences in mortality rates by type of health insurance. Both multivariable models included fixed effects for age group, urban or rural residence, and region of residence, as well as interactions with age. Data sets including values from the multiple imputations for missing risk factor data were included in these analyses

**Table 6:** Effect estimates for interacting variables in the cervical cancer models with imputed missing data (*n* = 5098)

	Associations between educational level or type of health insurance and cervical cancer mortality for each age group (years)											
	20–24		25–29		30–34		35–39		40–44		45–49	
	IRR	(95 % CI)	IRR	(95 % CI)	IRR	(95 % CI)	IRR	(95 % CI)	IRR	(95 % CI)	IRR	(95 % CI)
Model 1												
Educational level												
No education vs. higher education	26.8	(6.65–108)	16.7	(7.93–35.1)	14.7	(9.50–22.6)	10.6	(7.42–15.1)	12.3	(8.90–17.1)	8.49	(6.38–11.3)
Primary vs. higher education	14.0	(5.69–34.6)	12.9	(7.96–21.0)	6.38	(4.61–8.83)	5.60	(4.22–7.43)	6.28	(4.72–8.36)	4.98	(3.87–6.39)
Secondary vs. higher education	3.46	(1.42–8.45)	3.69	(2.26–6.02)	3.15	(2.27–4.36)	2.81	(2.10–3.75)	2.88	(2.15–3.88)	2.15	(1.64–2.81)
No education vs. primary education	1.91	(0.57–6.36)	1.29	(0.69–2.42)	2.30	(1.61–3.27)	1.89	(1.43–2.49)	1.96	(1.57–2.45)	1.71	(1.39–2.09)
Model 2												
Type of health insurance												
No insurance vs. contributory insurance	1.55	(0.65–3.67)	2.54	(1.71–3.77)	1.67	(1.24–2.27)	1.71	(1.33–2.21)	1.90	(1.50–2.41)	2.51	(2.01–3.15)
Subsidised vs. contributory insurance	2.06	(1.08–3.94)	2.04	(1.48–2.81)	1.90	(1.51–2.40)	1.79	(1.46–2.19)	2.14	(1.77–2.58)	2.18	(1.82–2.60)
Special vs. contributory insurance	4.01	(1.13–14.3)	0.26	(0.04–1.91)	0.58	(0.27–1.26)	0.55	(0.29–1.02)	0.86	(0.57–1.29)	0.88	(0.60–1.28)
Subsidised vs. special insurance	0.51	(0.16–1.68)	7.76	(1.08–55.8)	3.26	(1.52–6.99)	3.28	(1.78–6.05)	2.49	(1.67–3.70)	2.47	(1.71–3.57)
No insurance vs. special insurance	0.38	(0.10–1.44)	9.66	(1.33–70.4)	2.87	(1.31–6.30)	3.14	(1.67–5.92)	2.21	(1.45–3.38)	2.86	(1.93–4.23)
No insurance vs. subsidised insurance	0.75	(0.36–1.57)	1.24	(0.88–1.76)	0.88	(0.67–1.16)	0.96	(0.76–1.21)	0.89	(0.72–1.11)	1.16	(0.94–1.42)

*IRR* Incidence rate ratios, *95 % CI* 95 % confidence intervals

Model 1 assessed differences in cervical cancer mortality rates by educational level and Model 2 evaluated differences in mortality rates by type of health insurance. Both multivariable models included fixed effects for age group, urban or rural residence, and region of residence, as well as an interaction with age. Data sets including values from the multiple imputations for missing risk factor data were included in these analyses

Similarly, the pairwise comparisons describing the IRR for CC mortality among educational levels and insurance types for each age group using the imputed data (Table 6) had the same direction and similar effect sizes to the estimates resulting from the complete case analysis (Table 2). Additionally, differences in mortality rates observed in the imputed data analysis between younger and older women for each level of education were similar using the complete case data analysis (Table 3). The model based on imputed data including the type of health insurance (Model 2) also explained a larger portion of the total variance in CC mortality (AIC = 1138) than the model including the level of education (Model 1) (AIC = 1178).

## Discussion

Cervical cancer is a preventable and, if diagnosed early, a treatable disease for many women [2]. Despite this, the results of the present study reveal that a third of the women who died from CC in Colombia during the period of study were between 20 and 49 years. The loss of these women has a considerable consequence to young families and an economic impact on the Colombian society. Deaths in women of reproductive age could reflect limitations in strategies and resources to prevent and treat CC, such as challenges in accessing CC screening previously reported in Colombia [30]. Screening can have an important impact on reducing CC deaths [31]; however, to make a meaningful improvement in CC survival, screening needs to be accompanied by adequate access to follow-up and treatment options [14, 32]. The results of our study suggest inequitable access to either or both CC diagnosis and treatment among young women in Colombia.

The present study considered differences in CC mortality for women between 20 and 49 years associated with educational level and type of health insurance. The direction and strength of these associations were robust regardless of whether complete case or multiple imputation analysis was used. This suggests that missing data did not result in meaningful selection bias or a substantial loss of precision in the results. The visual assessment of the pattern of missing data informed the choice of imputation method and suggested that the data were most likely missing at random [33].

We found differences in CC mortality according to educational level, where a relative gap in CC mortality was observed among women with limited or no education compared to women with higher education, especially in the youngest groups. Lack of education has been described as a factor that perpetuates a vicious circle by limiting access of individuals to crucial information to prevent diseases [30], access to health care [34], and the practice of individuals’ health care rights [30, 34]. Also, low levels of education have been associated with increased frequency of riskier behaviours [35]. Other studies in Colombia have evaluated the relationship between education and mortality from other causes [12, 13, 35]. Notwithstanding, the age groups considered in these studies differ from the target age groups used in our analysis and women with no education were not included as a category in the previous analyses. Moreover, an interaction effect between age and educational level has not been previously explored. Our results provide evidence for a social gradient in CC mortality based on educational level which has the greatest impact among the youngest women. This finding suggests that improving education in young women or developing specific programs to improve access for women with no education or primary education could potentially decrease CC mortality.

We also observed differences in mortality rates according to health insurance with some variations among age groups. In addition to the differences in mortality among women with no insurance and those with contributory or special insurance, the observation that having subsidised insurance does not decrease CC mortality compared to not having insurance suggests the existence of potential limitations in CC diagnosis and cancer care for those with subsidised insurance, which could be a result of differences in benefits available as compared to contributory insurance [10, 20]. Although not demonstrated, late diagnosis and limited access to cancer treatment options [9] are potential explanations for the differences in CC mortality rates observed according to type of health insurance.

Additionally, simply having health insurance does not guarantee access to health services [34, 36]. Others have reported that patients face multiple barriers to access health care in Colombia, such as distance to health care centres, lack of cultural appropriateness of the services provided, political inefficiency, lack of knowledge about patients’ health care rights, and administrative barriers imposed by health insurance companies [36]. Sanchez et al. [34] wrote that, in spite of being insured, patients need to pay out-of-pocket for health services or deal with unknown and complex administrative formalities imposed by health insurance companies, which delay the provision of diagnosis or treatment. Our finding that the models with insurance explained more variability in CC mortality than the models with education status, based on their AICs, suggest insurance programs should be also a priority target for interventions to decrease CC mortality in Colombia for women between 20 and 49 years of age.

We also found that mortality rates in urban areas were higher than in rural areas after accounting for the effects of age, region of residence, and type of insurance or educational level. This result differs from studies conducted in other countries, in which CC mortality rates are high in rural areas [37, 38]. However, our results coincide with a previous Colombian study that suggests high CC mortality rates in urban areas [11]. The authors of the earlier study indicated that urban and rural differences in mortality rates could be affected by under-recording of CC deaths in areas with high levels of rurality [11]. However, others have specified that under-registration is low in Colombia and should not greatly bias mortality results [13].

Another plausible explanation for the rural-urban differences found in our study could be that, once diagnosed with CC, women living in rural areas often move to urban regions to seek cancer treatment and follow-up in better equipped health care centres. Oncologic services are mainly concentrated in big Colombian cities [39], forcing many women to leave their homes. Furthermore, a qualitative study in Colombia indicated the use persuasive strategies by some health insurance companies to convince women to change their address for expediting their referral to oncologic centres located in bigger cities [34]. If changing addresses is a common practice, then, it could be difficult to obtain accurate variations in CC mortality between rural and urban areas or even among Colombian regions.

Furthermore, mortality rates were higher among women living in the Atlantic, Central, Pacific, and Amazon-Orinoquía compared to the Eastern region after accounting for other risk factors. This finding could be related to social problems reported in departments of these regions including poverty or inadequate living conditions [19, 40], lack of access to primary and secondary CC prevention [30, 41], or unequal distribution of health care providers [42]. This might be a further indicator of geographical and socioeconomic difficulties in accessing oncologic centres in Colombia. A study from the Colombian National Cancer Institute [9], the main cancer centre in the country, showed that 47 % of patients seen in the Institute reside outside the capital of Colombia.

Our study is the first assessing CC mortality in Colombia among women aged 20–49 years using multivariable regressions to control for confounding by multiple socio–demographic variables and for missing values. We identified that CC mortality varied by both level of education and type of health insurance according to age groups, incorporating for the first time women who had special health insurance and women who did not have education. We made use of multiple databases to obtain the population at risk stratified for the risk factors of interest in our analysis. To obtain more complete estimates of CC mortality, cases classified as unspecified malignant neoplasms of the uterus were reallocated as CC cases. Additionally, to decrease loss of information and prevent potential bias due to missing data, we computed our results using multiple imputations in addition to complete case analysis. There was no evidence of substantial bias in the estimates from the complete case analysis in this data set.

The source of denominator data was the most substantial limitation of this study. To examine the effect of education, type of health insurance, and urban or rural residence, we made use of the best data available to estimate the population at risk for each age group. The distributions of 20-to-49-years women surveyed in the 2010 NDHS data set were applied to 2009 population projections in Colombia. Given that the 2010 NDHS was self-reported data, the distribution of the socio-demographic variables used to obtain the population at risk in our study would be limited by the quality of the survey results. Challenges with estimating risk factor group-specific denominator data for less populated regions also limited our ability to look at the joint effect of education and insurance in these data.

## Conclusion

Gaps in CC mortality between women with limited-to-no-education and highly educated women were identified with the greatest disparity in the youngest age groups. We also identified that mortality rates were higher among older women. Women with contributory and special health insurance had lower mortality due to CC than women with subsidised or no health insurance. However, women with subsidised health insurance did not have significantly lower CC mortality rates than those with no insurance. This suggests the need to critically review access to diagnostic and treatment services for women served by the subsidised insurance plan. Information on type of insurance described more variation in CC mortality in the overall study population than education status after accounting for other risk factors such as age, rural and urban differences, and region of residence. However, education appeared to be a stronger individual risk factor when comparing mortality rates among the most and least educated women. The detection of inequitable relative risks for CC mortality in young women associated with a number of socio-demographic risk factors represents an opportunity to target efforts to evaluate and improve CC prevention, diagnosis, treatment, and follow-up. Additionally, our results can be used to develop and implement interventions to optimise the impact of both existing and new resources to prevent premature mortality due to CC in Colombia.

## Additional files

Additional file 1: Results of the final model using complete data including the interaction between age and educational level (.xls format). *Description of the file:* This file contains the estimates of the final model using complete data that assessed the association between educational level and CC mortality adjusting by rural or urban residence, region of residence, and age. (XLSX 11 kb) Additional file 2: Results of the final model using complete data including the interaction between age and type of health insurance (.xls format). *Description of the file:* This file contains the estimates of the final model using complete data that assessed the association between with type of health insurance and CC mortality adjusting by rural or urban residence, region of residence, and age. (XLSX 11 kb) Additional file 3: Results of the final model using imputed data including the interaction between age and educational level (.xls format). *Description of the file:* This file contains the estimates of the final model using imputed data that assessed the association between with educational level and CC mortality adjusting by rural or urban residence, region of residence, and age. (XLSX 11 kb) Additional file 4: Results of the final model using imputed data including the interaction between age and type of health insurance (.xls format). *Description of the file:* This file contains the estimates of the final model using imputed data that assessed the association between with type of health insurance and CC mortality adjusting by rural or urban residence, region of residence, and age. (XLSX 11 kb)

## Abbreviations

95 % CI: 95 % confidence interval
AIC: Akaike’s information criterion
CC: Cervical cancer
DANE: National administrative department of statistics of Colombia (Departamento Administrativo Nacional de Estadística)
IRR: Incidence rate ratio
NDHS: 2010 national and demographic health survey
